# A collaborative strategy with community pharmacists and physicians to improve patient experience and implement quality standards for patients with depression

**DOI:** 10.1016/j.rcsop.2022.100125

**Published:** 2022-03-10

**Authors:** Anastasia Shiamptanis, Jenn Osesky, Joanna de Graaf-Dunlop

**Affiliations:** a Ontario College of Pharmacists, Toronto, Canada and New Brunswick College of Pharmacists, Moncton, Canada; b Planning and Integration, Ontario Health, Canada; c Population Health & Value-Based Health Systems, Ontario Health, Canada

**Keywords:** Continuous quality improvement, Depression, Primary care, Community pharmacy

## Abstract

**Background:**

The experience for patients with mental health disorders may be negatively impacted by the barriers to care, such as low health care provider-to-population ratios, travel time to reach service providers, higher hospital readmission rates, and local demand for services, especially in suburban and rural areas.

**Objectives:**

The project aimed to design a model in which physicians and pharmacists collaborate to provide comprehensive care to patients with depression in two northern communities and improve the patient and provider experience.

**Methods:**

Pharmacists and primary care physicians developed a model in which patients starting on new antidepressant medications received regular follow-up care and education on adjunct therapies from the community pharmacists instead of the physician. The patient and provider experiences were measured through surveys.

**Results:**

Out of the 14 patients who completed the patient survey, 13 reported feeling more supported by receiving follow-up care from pharmacists. Out of the 5 providers who completed the provider survey, 4 reported that the physician-pharmacist collaboration and additional support were helpful to patients.

**Conclusion:**

Overall, the project positively impacted patient experience and providers perceived value in the shared-care model.

## 1 Introduction

Mental illness is reported to be experienced by 1 in 3 Canadians during their lifetime.^1^ Yet, patients with mental illness face ongoing challenges in receiving mental health care.^2^ They are often misdiagnosed and receive inappropriate or inadequate treatment.^3^^,^^4^ Readmission rates for mental health and addiction issues are significantly higher than other health issues.^5^ In northern regions in Ontario, Canada, readmission rates are the highest in the province.^6^ Patients in this region have reported negative experiences due to fragmented care.^7^ Some of the challenges that patients face in the northern regions are low health care provider-to-population ratios, travel time to reach service providers, local demand for services,^8^ and nonadherence to treatment.^9^

Collaboration between primary care physicians and pharmacists may help address some of these challenges. Pharmacists are accessible health care providers who can help increase health system capacity and support continuity of care. Considering that medications such as antidepressants are a cornerstone of treatment for depression, pharmacists are well-positioned to provide follow-up care and support around the effectiveness, safety, and use of medications.^15^ Regular follow-ups with a health care provider are particularly beneficial to patients with depression after starting a new antidepressant to support adherence. Common reasons for nonadherence to antidepressants include concerns about side effects, fears of addiction, belief that the medication will not work, lack of sufficient patient education, and lack of follow-up.^16^ Also, regular follow-ups provide more opportunities for patients to participate in their care, which has been shown to improve adherence in patients with depression.^17^ By effectively engaging pharmacists within the health care system, patients can have timely access to quality care and improve the overall patient experience.

Patient experience is an important measure of health care quality and provides useful information about how patients assess their care.^10^^,^^11^ Additionally, given the positive correlation between patient experience and health care outcomes, efforts to measure the patient experience can serve as a meaningful measure of quality.^12^^,^^13^ One method for measuring experience is through a patient survey tool, which collects information about the overall patient experience, and can measure incremental changes, such as a new method for delivering care.^14^

Improving patient experience is a key priority for Ontario.^18^ Ontario Health (OH) developed quality standards for Major Depression and other conditions based on evidence and expert consensus.^19^ The quality standards are intended to inform best practices and help health care organizations measure, assess, and improve their performance in caring for patients. Continuous Quality Improvement (CQI) is a method to implement measurable quality improvement in patient experience and outcomes.^20^ Pharmacists have demonstrated a capacity to implement CQI strategies to deliver meaningful change.^21^ However, strategies to implement the OH quality standards for depression are lacking. This project fills this gap. The project aimed to improve the patient experience by enhancing physician-pharmacist collaboration and implementing quality standards using CQI.

A CQI project based on the OH quality standards for depression was designed in this project. One of the key features of the CQI project was the collaboration between primary care physicians and pharmacists to provide longitudinal care during regular follow-ups. The project was conducted in two northern communities in Ontario (Sudbury and Espanola). Patient surveys were utilized to evaluate the overall patient experience. The experience of pharmacists and physicians was also measured via provider surveys to gain their perspective on the collaborative approach.

## 2 Methods

Together with a team of two primary care physicians and six pharmacists, all practicing in the two northern communities, a CQI project was developed that aligned to the OH quality standards for Major Depression.^19^ One component of the CQI project was the practice model, designed to improve the patient experience and increase health system capacity for mental health in the community. Training sessions and support on CQI were provided to the team throughout the project duration.

### 2.1 Principles

The team established the following six principles to underpin the CQI project:

1. *Patient-centred:* The CQI project will be modeled with the patient at the centre of the health care delivery system. A strong primary care foundation, as well as collaboration and communication within and between primary care practitioners and community pharmacists along the continuum, are essential to achieving patient-centered care.
2. *Practicing to optimal scope*: The CQI project will encourage all professionals to work to their optimal scope of practice. Clinicians will be supported to practice in an environment that allows them to fully utilize their knowledge, skills, and expertise to benefit patients and the health care system.
3. *Quadruple aim*: The project will take a population-based health approach, enhancing the experience and outcomes for patients, improving health system efficiency, and will seek to improve the experience of those providing the care.^22^
4. *Shared Accountability*: All professionals and organizations involved in the CQI project will be responsible for ensuring the best outcomes for their patients. A structured approach to sharing outcome measures will support shared accountability.
5. *Scalable:* To spread to a wider range of patients, the model will be reproducible and portable.
6. *Sustainable:* The model's infrastructure will be able to be supported and maintained.

### 2.2 Quality statements

The team reviewed all statements within the Ontario Health quality standard for Major Depression and identified the two highest impact areas for a joint physician and community pharmacist intervention^19^:

• Quality Statement # 5: Adjunct Therapies and Self-Management: People with major depression are advised about adjunctive therapies and self-management strategies that complement antidepressant medication or psychotherapy.
• Quality Statement #6. Monitoring for Treatment Adherence and Response: People with major depression are monitored for the onset of, or an increase in, suicidal thinking following initiation of any treatment. People with major depression have a follow-up appointment with their health care provider at least every 2 weeks for at least 6 weeks or until treatment adherence and response have been achieved. After this, they have a follow-up appointment at least every 4 weeks until they enter remission.

### 2.3 Practice model

**Fig. 1:**
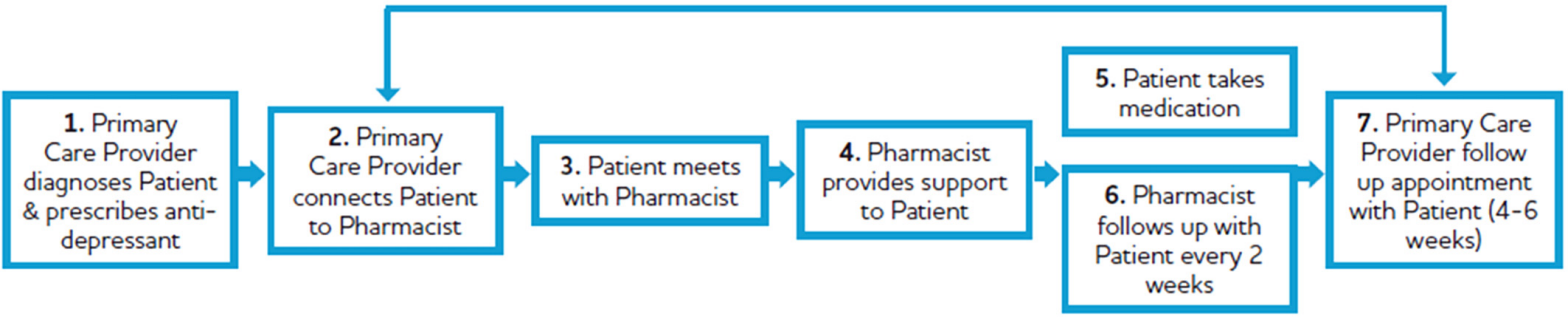
Practice model.

In the practice model developed, the primary care physicians asked patients starting on new antidepressant medication to follow up with their community pharmacist every two weeks. Patients who were newly diagnosed with depression or changed medication therapy were eligible to participate. Patients with suicidal ideation were excluded. Pharmacists supported adherence to therapy and reinforced education on adjunct therapies until the follow-up appointment with the primary care physician (Step 7 in Fig. 1). The appointment with the primary care physician was usually 6 weeks after starting the new antidepressant medication. A key step of the practice model was that the follow-ups every 2 weeks (Step 6 in Fig. 1) were completed by the pharmacist instead of the primary care physician to enhance health system capacity. Each patient would receive at least two follow-ups with their pharmacist. After establishing the model, the team invited other primary care physicians and community pharmacists practicing in the two northern communities to participate.

Through active outreach, the team recruited 7 physicians and 11 pharmacists in the two communities. A project toolkit was developed to support participating pharmacists and physicians, which included a description of the practice model, key resources for adjunct therapies, templates for communication between physicians and pharmacists, information on local mental health care resources, the PHQ-9 questionnaire, and a copy of the patient survey.^23^ Although the PHQ-9 is a tool to measure the response to treatment for patients with depression, it was not a requirement to complete. Instead, it was used as a tool to guide discussions between the pharmacist and the patient. The toolkit provided consistent information to all participating pharmacists and physicians to facilitate fidelity of the model and standardized the adjunct therapies provided to patients.

Throughout the project period, a plan-do-study-act (PSDA) approach was applied. Regular meetings were held with the participants to discuss any barriers and enhancements to the project. The meetings served as a forum to implement improvements and modifications based on timely feedback and observations. For example, an adjustment was made to enable pharmacists to identify patients and enroll them in the project to broaden recruitment. The group determined that recruitment through physicians limited patient enrollment. Due to time constraints, it was not always feasible to introduce the project during physician visits.

### 2.4 Study outcomes

The primary outcome was patient experience, and the secondary outcome was provider experience.

### 2.5 Data collection

For the primary outcome, a patient experience survey was created using the experience-based co-design (EBCD) toolkit.^24^ EBCD focuses on patient experience and emotions and can provide rich insights into patients' experiences. Emotionally significant points were identified to design an emotional map with positive and negative feelings. Emotional mapping questions add a more qualitative dimension to the analysis and highlight changes in the patient's emotions.^25^

The patient experience survey was conducted at the 6-week follow-up appointment by the pharmacist or the primary care physician. The survey consisted of two questions and an emotional mapping questionnaire.

The two questions asked were “I felt that the additional supports provided to me (i.e., to help me with my medication and/or information about other therapies) were helpful to me” and “If friends and/or family were in a similar situation, I would recommend the additional supports I received to them.” The two questions were answered based on a 5-point Likert scale with responses ranging from ‘strongly agree’ to ‘strongly disagree.’

For the emotional mapping question, patients were asked to select from a list of 12 words the feelings they had *when they started taking antidepressant medication* and *when they received additional support with their antidepressant medication*. The word selection consisted of 7 ‘positive’ words and 5 ‘negative’ words. When presented to the patients, the 12 words were not stratified into positive and negative words but instead were arranged in the following assorted order: safe, supported, concerned, calm, disappointed, worried, informed, appreciated, knowledgeable, frustrated, understood, uncomfortable.

During the follow-ups, pharmacists assessed medication adherence and response to therapy, provided clinical activities (such as identifying drug-related problems and giving pharmaceutical opinions to physicians^26^), and provided education on adjunct therapies where appropriate. Adjunct therapies, such as healthy sleep habits, healthy eating habits, physical activity, yoga, and light therapy, can be effective complements to antidepressant medication, potentially resulting in faster improvement and fewer residual symptoms.^27^

For the secondary outcome, a provider experience survey was conducted. Providers were invited to complete the survey once the CQI project was completed. The survey consisted of four questions rated using the same 5-point Likert scale as the patient experience survey. The four questions asked were, “I felt that the additional collaboration between primary care provider and pharmacist was helpful to the patients who participated,” “I feel that the additional support for medication adherence and response was helpful to the patients who participated,” “I feel that the additional supports re: adjunct therapies were helpful to the patients who participated” and “I would recommend this project to my colleagues.”

### 2.6 Data analysis

Two methods were used to analyze data. First, questions based on the Likert scale were analyzed by aggregating the responses according to strongly agree/agree, neither agree nor disagree, and strongly disagree/disagree. Second, the emotional mapping was analyzed by stratifying into ‘positive’ and ‘negative’ words and measuring their frequency.

## 3 Results

The project started in February 2019 and ended in December 2019. During the 11-month project, 35 patients who started on new antidepressant medication were offered enrollment. Of the 35 patients, 30 (85.7%) agreed to participate, and 5 (14.3%) declined.

The 30 participants received, on average, 2 follow-ups. During the follow-ups, pharmacists assessed medication adherence and response to therapy for all 30 patients. Of the 30 patients, pharmacists identified drug-related problems in 13 (43.3%) patients and provided pharmaceutical opinion/intervention to 10 (33.3%) patients. Physicians accepted all recommendations made by pharmacists. In one case, a pharmacist recommended titrating a dose of the antidepressant more slowly to reduce side effects. Additionally, on average, pharmacists provided support on 1 adjunct therapy per patient. The type of adjunct therapies provided was determined based on individual patient factors. Of the 30 patients, 12 (40.0%) received additional support on healthy sleep habits, 8 (26.6%) on physical activity, 5 (16.6%) on nutrition and 1 (3.3%) on yoga. Table 1 reports the activities provided by pharmacists.

**Table 1:** Activities provided by the pharmacists during follow-ups.

Activity	Patients who received support n(%)
Medication adherence/response to therapy	30 (100)
Drug-related problems	13 (43.3)
Pharmaceutical opinion/intervention	10 (33.3)
*Adjunct support*	
Healthy Sleep Habits	12 (40)
Physical Activity	8 (26.6)
Healthy Eating Habits/Nutrition	5 (16.6)
Yoga	1 (3.3)
Light Therapy	0 (0.0)

Of the 30 participants, 14 (46.7%) completed the patient experience survey. For the first and second questions, 13 patients (92.9%) reported that they “strongly agree/agree,” and 1 patient (7.1%) selected “neither agrees nor disagrees.” Table 2 summarizes the responses.

**Table 2:** Patient experience survey results.

Question	Strongly agree/ agree (n)	Neither agree nor disagree (n)	Disagree/ strongly disagree (n)
I felt that the additional supports provided to me (i.e., to help me with my medication and/or information about other therapies) were helpful to me.	13	1	0
If friends and/or family were in a similar situation, I would recommend the additional supports I received to them.	13	1	0

The emotional mapping was analyzed to determine any trends in negative and positive responses. On average, patients selected 3–4 words to describe their emotions. Of the 14 patients, 6 patients (42.9%) selected at least one negative word when asked how they felt *when they started taking their antidepressant*. The most common negative word was “worried” (*n* = 3). In contrast, no patient selected a negative word when asked how they felt *after receiving additional support from the pharmacist*. Also, the frequency of the positive words increased after the patients obtained additional support. The most common positive words were “supported” (*n* = 13) and “safe” (*n* = 9), and the words with the largest increase in frequency were “supported” and “appreciated.” Table 3 provides the frequency of the positive and negative words.

**Table 3:** Frequency of positive and negative words selected in the emotional mapping questions.

	When I started taking antidepressant medication (n)	When I received additional support to help me with my antidepressant medication (n)
*Positive*		
Safe	7	9
Supported	9	13
Calm	2	1
Informed	7	7
Appreciated	4	8
Knowledgeable	3	4
Understood	3	6

*Negative*		
Concerned	2	0
Disappointed	0	0
Worried	3	0
Frustrated	1	0
Uncomfortable	2	0

Of the 18 providers (7 physicians and 11 pharmacists) invited to complete the provider experience survey, 5 providers (3 physicians and 2 pharmacists) completed it. The 2 pharmacists “strongly agree/agree” to all four questions. Of the 3 physicians, 2 “strongly agree/agree” that the *collaboration between providers was beneficial to patients* and *would recommend the project to colleagues*. There was 1 physician who “strongly agree/agree” that the *support for medication adherence* and *adjunct therapies* was helpful to patients. Table 4 summarizes the responses.

**Table 4:** Provider experience survey.

Question	Strongly agree/agree (n)	Neither agree nor disagree (n)	Disagree/strongly disagree (n)
I felt that the additional collaboration between the primary care provider and pharmacist was helpful to the patients who participated	4 (2 pharmacists, 2 physicians)	1 (1 physician)	0
I feel that the additional support for medication adherence and response was helpful to the patients who participated	3 (2 pharmacists, 1 physician)	2 (2 physicians)	0
I feel that the additional supports re: adjunct therapies were helpful to the patients who participated	3 (2 pharmacists, 1 physician)	2 (2 physicians)	0
I would recommend this project to my colleagues	4 (2 pharmacists, 2 physicians)	1 (1 physician)	0

## 4 Discussion

This project found that the patients' experience improved with the practice model. The patients viewed the support provided by pharmacists as valuable to their overall experience. Pharmacists contributed to patient care through various clinical activities. In addition to implementing best practices to support medication adherence (through patient follow-up) and self-management (through adjunct therapies and mental health supports), pharmacists optimized medication therapy by identifying and addressing medication-related problems in collaboration with the physician. Additionally, the negative feelings experienced by patients at the time of starting the medication disappeared after patients received follow-ups from the pharmacist, demonstrating the benefits of continued support.

The role of pharmacists in this project is similar to that reported by Haslam et al.^28^ in a study that sought to characterize the patient care activities of pharmacists in the Bloom Program, a community pharmacy-based program that increases and enhances mental health and addictions services for patients in Nova Scotia.^29^ In their study, medication management was the most common activity, and other activities included education, collaboration, social support, navigation, and resource support.

Furthermore, the provider experience survey findings suggest that the physicians and pharmacists believed there was value in the additional support provided to patients. The pharmacists appeared to be slightly more positive than the physicians that the project was helpful to patients. During the project, pharmacists interacted more frequently with patients, which may have influenced their more positive perception of patient satisfaction.

The project also demonstrated that this collaboration facilitated the implementation of best practices. This project builds upon existing literature in which pharmacists have applied CQI with primary health care teams. In a study conducted by Raiche et al.,^30^ pharmacists in primary care led a CQI initiative to optimize medication therapy for patients on proton-pump inhibitors according to evidence-based guidelines. Similar to the study in this paper, in addition to implementing best practices, the CQI initiative provided an opportunity for community pharmacists to foster collaborative relationships with physicians, creating more connections across the health care system for patients.

Additionally, this project demonstrated the value of involving pharmacists as part of the broader primary health care team. Physicians and pharmacists participated in a debriefing session to synthesize learnings at the end of the project. A foundation to effective collaboration is establishing trusting relationships among providers facilitated through communication. Legacy methods, such as fax, were seen as a barrier by some physicians. While others utilized a more streamlined approach, such as using secure web-based means or electronic patient care records, to foster more effective communication. The group emphasized the value of standard tools and templates available in the project toolkit as an enabler to support implementation and consistent communication. The group also recognized the importance of change management and engaging clinical champions early in the process to leverage their expertise and influence and sustain project momentum. Physicians suggested involving other primary care professionals, such as nurses and allied health staff, in future CQI projects to facilitate greater uptake and enrollment. The pharmacists reported they could sustain this model and embed this into their practice. Lessons learned from the group may help other teams implement a pharmacist-physician collaborative CQI project to support patient experiences with conditions like COPD and diabetes.

### 4.1 Limitations

This project was designed as a CQI initiative, and the results should be interpreted accordingly. As such, there was not a control group to compare the outcomes measures or establish a baseline. A future project may enrich the understanding of the impact of this work. Additionally, the PHQ-9 was an optional tool rather than a required tool. The use of validated tools may enhance the generalizability of the results.

Three of the five providers who completed the provider experience survey created the practice model, potentially introducing bias and skewing towards more favourable responses. Further, patients that agreed to participate may have been more willing to accept additional support and, therefore, may positively skew patient experience. Additionally, patients with suicidal ideation were not enrolled due to the need for more close physician supervision, introducing potential recruitment bias.

## 5 Conclusion

Continuous quality improvement was used as a framework to establish a collaborative model between physicians and community pharmacists to implement evidence-based standards in practice and improve the experience for patients with depression. The collaborative approach to care reinforced the role of pharmacists in improving the quality of care and strengthening access to health care. Pharmacists were well-positioned to provide follow-up and monitoring in the community. A similar approach may be taken to address other local health care needs. Larger studies that build on this approach may further elucidate the impact on patient and system outcomes.

## Funding

No external sources of funding were used for the conduct of this study or the writing, correction, and submission of this article.

## Declaration of Competing Interest

The authors declare that they have no known competing financial interests or personal relationships that could have appeared to influence the work reported in this paper.
